# DIAN‐TU‐001 Trial (Tau NexGen) Rationale and Enrollment Experience

**DOI:** 10.1002/alz70859_105298

**Published:** 2025-12-25

**Authors:** Lon S. S Schneider, Jorge J. Llibre‐Guerra, David B. Clifford, Susan Mills, Guoqiao Wang, David Li, Tammie L.S. Benzinger, Brian A. Gordon, Eric McDade, Shobha Dhadda, Michael C. Irizarry, Larisa Reyderman, Randall J. Bateman

**Affiliations:** ^1^ Alzheimer’s Disease Research Center, Keck School of Medicine, University of Southern California, Los Angeles, CA USA; ^2^ Banner Alzheimer's Institute, Phoenix, AZ USA; ^3^ Keck School of Medicine of USC, Los Angeles, CA USA; ^4^ Department of Neurology, Washington University School of Medicine, St. Louis, MO USA; ^5^ Washington University in St. Louis, School of Medicine, St. Louis, MO USA; ^6^ Washington University in St. Louis School of Medicine, St. Louis, MO USA; ^7^ Washington University School of Medicine, St Louis, MO USA; ^8^ Eisai Inc., Nutley, NJ USA; ^9^ Washington University School of Medicine, St. Louis, MO USA

## Abstract

**Background:**

Alzheimer's disease (AD) is characterized by the sequential but overlapping accumulations of amyloid‐beta plaques and tau neurofibrillary tangles prior to symptom onset. Tau plays a fundamental role in microtubule stabilization, but tau species containing microtubule binding region (MTBR) domains can form toxic insoluble aggregates under pathological conditions. Therapies targeting tau MTBR may disrupt tau aggregation and prevent initiation or attenuate neurodegeneration. This arm of the DIAN‐TU‐001 Trial is designed to investigate tau‐targeting therapies to help determine the role of tau in disease biology and progression in dominantly inherited Alzheimer's disease (DIAD). The goal is to investigate potential benefits of the anti‐tau therapy etalanetug (E2814), a novel monoclonal antibody that binds to the MTBR, with the anti‐amyloid antibody, lecanemab, given as background therapy.

**Method:**

The DIAN‐TU‐001 Tau NexGen Trial (NCT05269394) is a phase II/III randomized, double‐blinded, placebo‐controlled trial for participants with a known pathogenic DIAD mutation in PSEN1, PSEN2, or APP genes, who are between −10 and +10 years from their expected age of symptom onset (EYO) and received a CDR score of 0 or 0.5 to 1. Mutation‐positive participants were enrolled in each of 2 cohorts (Cohort 1: Symptomatic Population [CDR=0.5 or 1], n=97; Cohort 2: Asymptomatic Population [CDR=0];n=100). Cohort 1 will receive open‐label lecanemab at Week 0 and then be randomized to receive concurrent E2814 or placebo at week 24; Cohort 2 will be randomized to receive E2814 or placebo at Week 0 and then receive concurrent open‐label lecanemab from Week 52.

**Result:**

The primary objective of this arm is to determine whether etalanetug is superior to placebo, when administered with lecanemab, in change from Week 24 to Week 104 and Week 208 in tau spread as measured by tau PET in Cohort 1. Additional objectives include evaluating etalanetug vs placebo with CDR‐SB (Cohort1), CSF ptau217)/total‐tau (Cohort2), and other cognitive, clinical, imaging, fluid biomarker, and safety outcomes assessed at randomization/baseline and specific follow‐up time points throughout the trial. Amyloid reduction and safety assessments when lecanemab was administered alone in Cohort 1 were evaluated.

**Conclusion:**

This trial will investigate potential benefits of anti‐tau etalanetug with lecanemab, given as background therapy.

